# Effects of polymorphic variation on the thermostability of heterogenous populations of CYP3A4 and CYP2C9 enzymes in solution

**DOI:** 10.1038/s41598-018-30195-1

**Published:** 2018-08-08

**Authors:** Lauren B. Arendse, Jonathan M. Blackburn

**Affiliations:** Institute for Infectious Disease & Molecular Medicine, Department of Integrative Biomedical Sciences, Faculty of Health Sciences, University of Cape Town, Observatory, 7925 South Africa

## Abstract

The effect of non-synonymous single nucleotide polymorphisms (SNPs) on cytochrome P450 (CYP450) drug metabolism is currently poorly understood due to the large number of polymorphisms, the diversity of potential substrates and the complexity of CYP450 function. Previously we carried out *in silico* studies to explore the effect of SNPs on CYP450 function, using *in silico* calculations to predict the effect of mutations on protein stability. Here we have determined the effect of eight CYP3A4 and seven CYP2C9 SNPs on the thermostability of proteins in solution to test these predictions. Thermostability assays revealed distinct CYP450 sub-populations with only 65–70% of wild-type CYP3A4 and CYP2C9 susceptible to rapid heat-induced P450 to P420 conversion. CYP3A4 mutations G56D, P218R, S222P, I223R, L373F and M445T and CYP2C9 mutations V76M, I359L and I359T were destabilising, increasing the proportion of protein sensitive to the rapid heat-induced P450 to P420 conversion and/or reducing the half-life of this conversion. CYP2C9 Q214L was the only stabilising mutation. These results corresponded well with the *in silico* protein stability calculations, confirming the value of these predictions and together suggest that the changes in thermostability result from destabilisation/stabilisation of the protein fold, changes in the haem-binding environment or effects on oligomer formation/conformation.

## Introduction

Cytochrome P450 (CYP450) enzymes, arguably nature’s most versatile catalysts, are a superfamily of haem-thiolate proteins found across all lineages of life^1^. CYP450s play a key role in human drug metabolism, oxidising 70–80% of pharmaceutical drugs in phase I drug metabolism^2^. While there are more than 57 different CYP450 enzymes in humans, only a small number of highly polymorphic isoforms are responsible for the majority of drug metabolism^2^. The occurrence and frequency of polymorphic variation varies between ethnic groups and has been shown to affect drug response^3^. Variant alleles include deletions, insertions, copy number variants and single nucleotide polymorphisms (SNPs), both in the coding and non-coding regions of the genes, which can alter CYP450 expression levels as well as protein function^4^. Over 100 non-synonymous single amino acid substitutions have been reported for isoforms CYP3A4 and CYP2C9 alone^5,6^; these two isoforms are jointly responsible for nearly half of CYP450 mediated drug metabolism^2^. The large number of polymorphisms and potential drugs, together with the observation that the effect of SNPS can be substrate specific^7–10^, means that the phenotypic impact of the majority of variants is still poorly understood and difficult to predict.

There are now around 800 published CYP450 X-ray crystal structures, including well over 100 human CYP450 structures crystallised in the presence and absence of a range of ligands. CYP450s have a highly conserved globular fold, typically made up of 13 α-helices and 4 to 5 β-sheets enclosing a large buried hydrophobic active site^11^. The enzyme comprises a relatively flexible domain on the distal side of the protein, primarily responsible for substrate recognition and binding; a more rigid haem-binding core; and a domain with intermediate flexibility on the proximal side of the protein that provides a binding site for the redox partner - responsible for transferring electrons to the haem iron during the catalytic cycle - in close proximately to the catalytic centre^12^. The haem-binding regions are generally conserved between CYP450s while the substrate recognition regions are more variable^13^. There are a number of key conserved features found in all CYP450s: the I-helix catalytic groove^11^ which plays an important role in electron transport^14,15^ and forms the oxygen binding pocket^16^; the K-helix core stabilising motif comprising the invariant EXXR motif which interacts with a conserved Arg/His residue in the meander region, forming the ERR triad^17^; and the Cys-pocket surrounding the cysteine residue that co-ordinates the haem ion.

Most human CYP450s are microsomal CYP450s bound to the endoplasmic reticulum membrane by an N-terminal anchor. While CYP450s have traditionally been regarded as monomers, there is increasing evidence that cross-talk occurs between multiple CYP450 isoforms within the membrane via homo- and hetero-oligomerisation^18,19^. Atypical kinetic profiles are commonly observed for drug metabolising CYP450 isoforms^20,21^ and crystal structures have confirmed that multiple ligands can bind within the large flexible active sites of these enzymes. In addition, substrate binding has been described as a multistep process and residues on the periphery of the catalytic binding site are thought to form an initial binding site important for substrate specificity in some isoforms^22–24^.

Single amino acid substitutions can affect haem binding, substrate access and binding, interactions with redox partner cytochrome P450 reductase (CPR), oligomerisation and/or the conformation and structural stability of the enzyme. Effects of amino acid substitutions on protein structure and activity can be manifested in a variety of ways. In addition to direct effects on key interactions with co-factors, ligands and protein binding partners, mutation can also have indirect effects on protein function which are far more difficult to predict. Mutations affecting stability can lead to the formation or disruption of secondary structure elements and both local and long-range conformational changes, which in turn can change the topology, solvation and accessibility of the active site. In a previous study, we combined *in silico* protein stability calculation with structure-function relationships to predict the phenotypic effect of SNPs in the major drug metabolising CYP450 isoforms^25^. Site Directed Mutator (SDM) software^26^ was used to predict the effect of mutations on the stability of the apoprotein structure. This software uses environment-specific substitution frequencies within homologous protein families to access the probability of a given residue being substituted by another one, given the amino acids local structural environment. The algorithm uses these environment specific substitution tables to calculate a stability score, by predicting the difference in free energy between a wild-type and mutant protein, by analogy to a reversible folding-unfolding thermodynamic cycle. To account for direct effects of mutations on protein function, a cytochrome P450 SNP map was created which combined information from previous functional studies to delineate regions important for substrate recognition^27,28^, haem binding, interactions with CPR^29–34^ and residues implicated in the gating of substrate and product access/egress tunnels^35–37^. The combination of this information provides a useful tool for predicting the effect of mutations on protein function and for prioritising variants for *in vitro* testing^25^. CYP450 function is affected *in vivo* by a range of factors, including interaction with the membrane, redox partners and other CYP450 molecules, as well as the diverse range of substrates that could be affected by SNPs in a substrate specific manner. We have previously mapped the dynamic substrate and solvent access channels, as well as redox partner binding sites, in CYP450s^25,34,36^; from these and other data, it is clear that whilst the haem environment of CYP450s is primarily responsible for the high-energy iron(iv)-oxo chemistry, the relationship between thermostability and catalytic function is complex and difficult to directly correlate. Despite this, in our previous study we found that SNPs predicted to alter protein stability or located in regions important for catalytic function are more likely to have effects on CYP450 function, based on available *in vitro* activity data^25^.

The aim of this study was to test our previous *in silico* stability prediction *in vitro*, using a selection of recombinantly expressed CYP3A4 and CYP2C9 mutants. The 8 CYP3A4 and 7 CYP2C9 variants tested included mutations previously predicted to be neutral, stabilising and destabilising, falling within both functional and undefined regions of the protein structure^25^.

## Results

### CYP450 expression and spectral analysis

**Figure 1:**
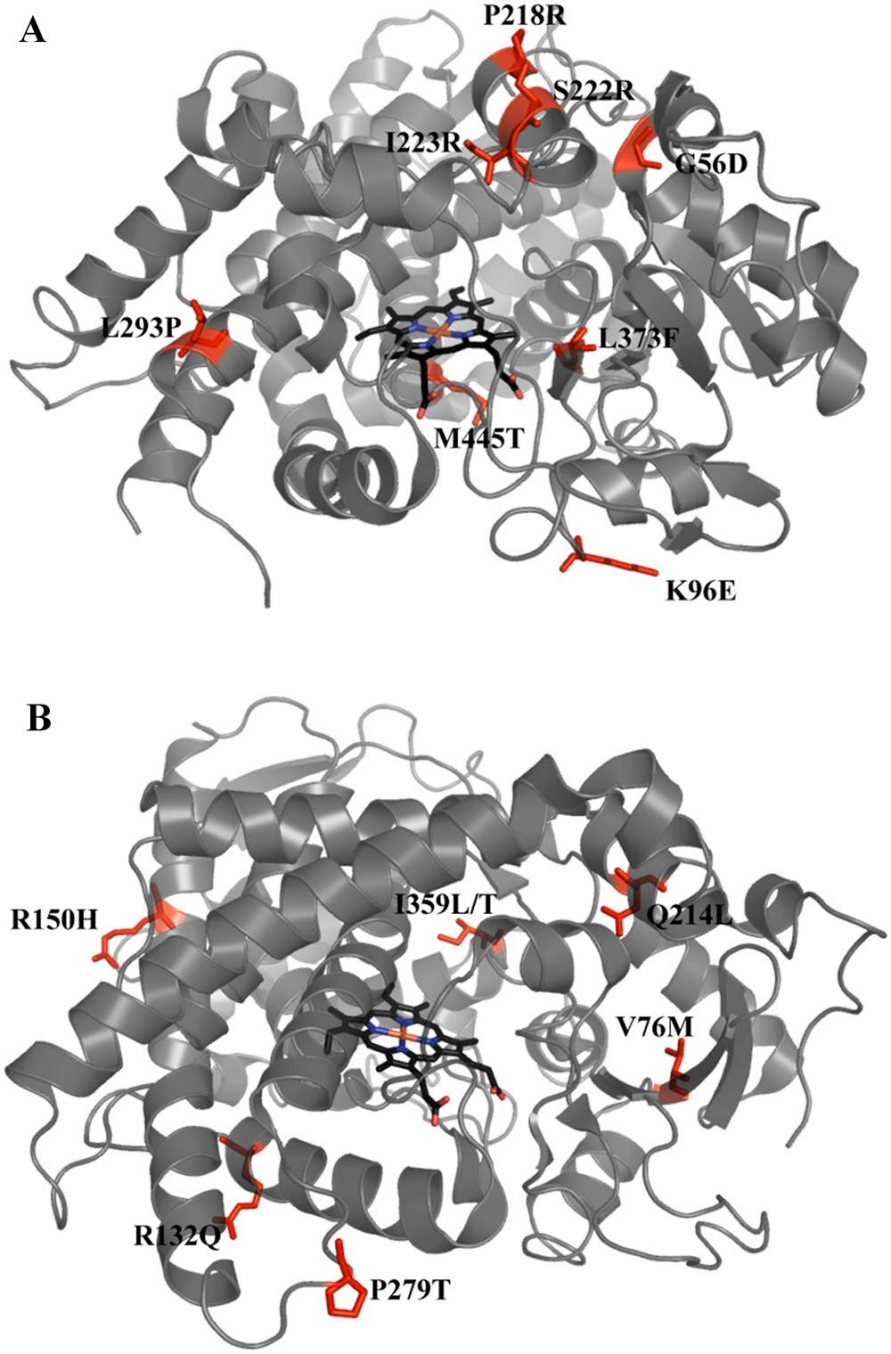
Positions of amino acid substitutions in CYP3A4 and CYP2C9 structures. (**A**) CYP3A4, PDB structure 1TQN, and (**B**) CYP2C9, PDB structure 1OG2, are shown in grey with haem groups in black. The positions of mutations are labelled and indicated in red.

The positions of the CYP3A4 and CYP2C9 variants selected for *in vitro* testing are shown in Fig. 1. Recombinant wild-type (WT) and variant His-tagged CYP3A4 and CYP2C9 proteins were expressed in *E. coli* and purified using immobilized metal affinity chromatography. The specific holoprotein content of each CYP3A4 and CYP2C9 variant protein sample was determined using carbon monoxide (CO) P450 spectral assays^38,39^. This assay distinguishes between two forms of the holoprotein: the P450 form and the P420 form. The reduced ferrous form of the haem ion binds CO, yielding an absorption peak at 450 nm, provided the haem group is correctly incorporated into the CYP450, providing a measure of the active protein in the sample (although technically the enzyme is rendered inactive on coordination to CO) (Fig. S1). In contrast, the inactive P420 form, yields an absorption maximum at 420 nm when bound to CO. The P450 to P420 conversion is associated with disruption to the haem-binding environment resulting in loss of the cysteine thiolate - haem ion coordination.

**Figure 2:**
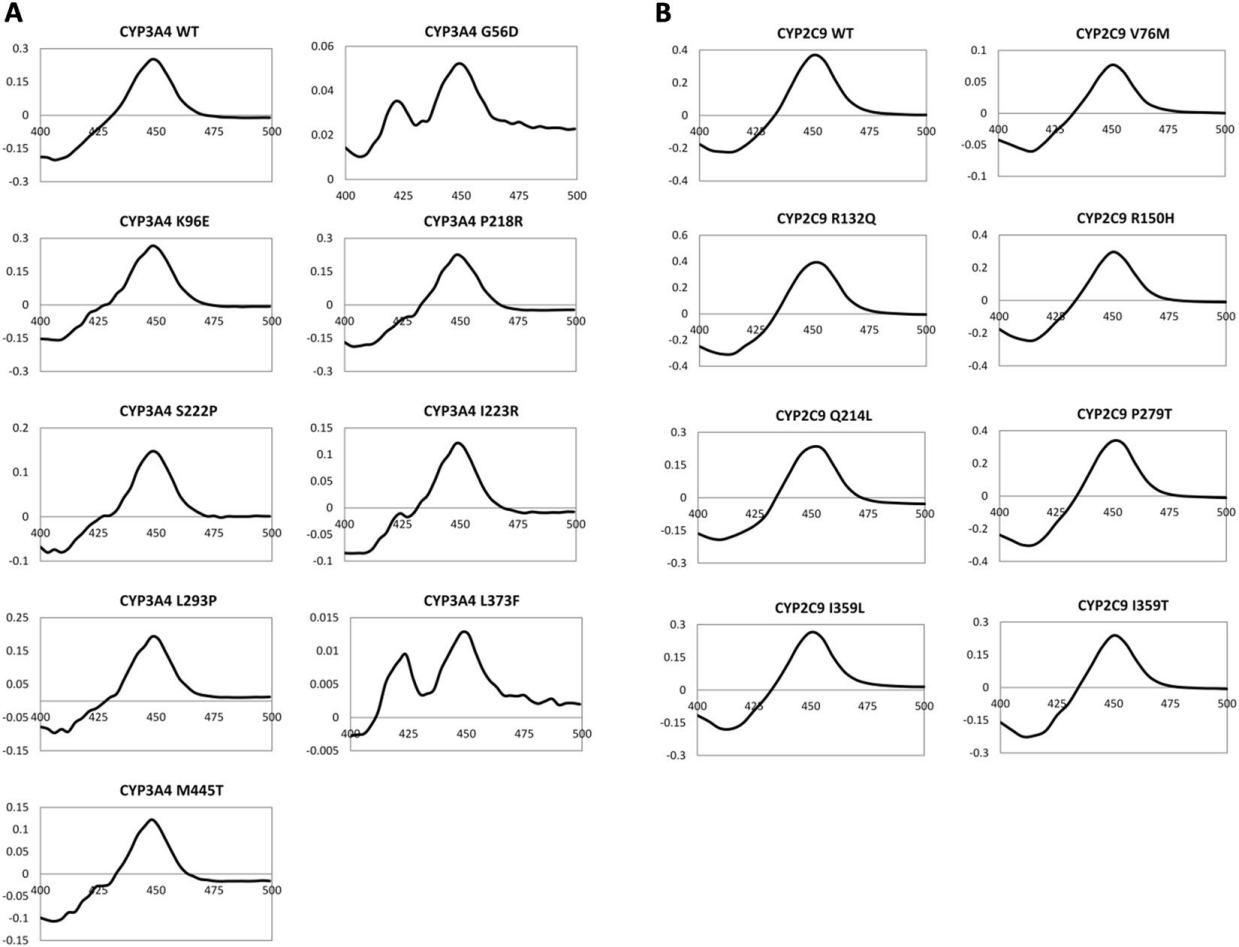
Ferrous CO vs. ferrous difference spectra. Spectra for (**A**) CYP3A4 and (**B**) CYP2C9 wild-type and variant protein samples. Wavelength is shown on the x-axis and difference in absorbance is shown on the y-axis.

The P450 form of the protein was detected, at varying levels, in all CYP3A4 and CYP2C9 protein samples; while the inactive P420 form was present in all CYP3A4 protein samples, including WT, but absent in all CYP2C9 protein samples (Fig. 2 and Supplementary Fig. S2). All CYP3A4 variants showed elevated P420 to P450 ratios compared with the WT protein. G56D and L373F variants had the lowest P450 content – only 12% and 4% of WT - and the highest P420 to P450 ratios, suggesting that these mutations decrease protein stability and/or haem incorporation. The P450 content for CYP2C9 WT and variant samples was generally higher than for the CYP3A4 protein samples. The V76M variant had the lowest P450 content of the CYP2C9 variants (20% of WT).

### Thermostability CO P450 spectral assays

**Figure 3:**
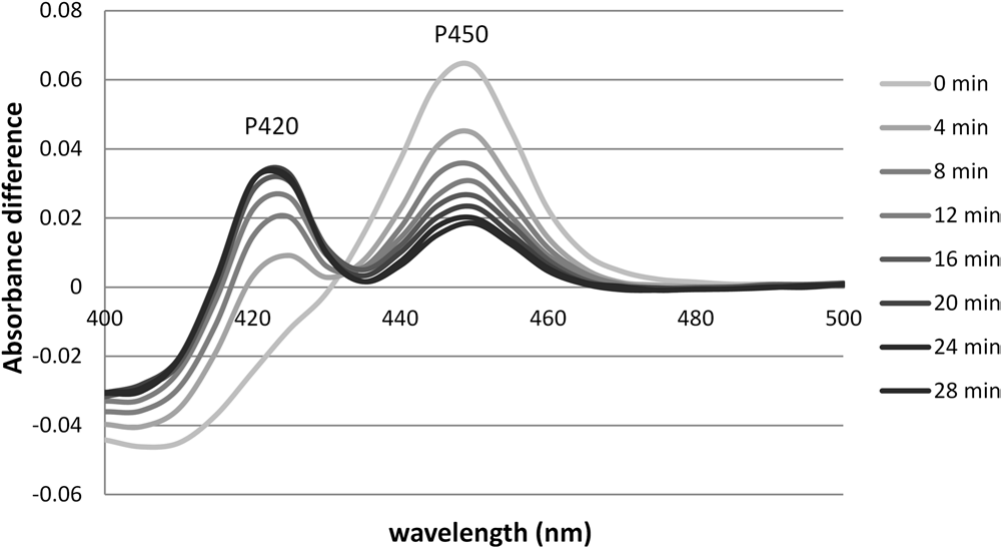
Ferrous CO vs. ferrous difference spectra for CYP3A4 WT thermostability assay. The difference spectra show a decrease in P450 and an increase in P420 with time. Assay was carried out at 34 °C and absorbance spectra measured every 4 minutes are shown.

To test the thermostability of the CYP3A4 and CYP2C9, WT and variant proteins, CO P450 difference spectra were used to measure the decrease in P450 content at an elevated temperature as a function of time. When the CYP450 protein samples were incubated at an elevated temperature, the peak at 450 nm shifted to 420 nm indicating the thermal conversion of active P450 into inactive P420 (Fig. 3). Different temperatures were tested to ensure the decrease in P450 could be monitored at a constant temperature over a 30-minute period: 34 °C was chosen for CYP3A4 proteins and 48 °C for the more stable CYP2C9 proteins. CYP3A4 and CYP2C9 WT protein samples both showed an exponential decrease in P450 content, although the graphs did not tend to zero, suggesting either the presence of a stable P450 sub-population or background signal. When the temperature was elevated further, up to ~60 °C, in both cases the peak at 450 nm disappeared rapidly followed by a decrease in P420 until no peaks were visible. This data implies that the asymptote - or ‘low plateau’ - was a result of a more stable P450 sub-population rather than background signal.

### Comparing the thermostability of CYP3A4 polymorphic variants

**Figure 4:**
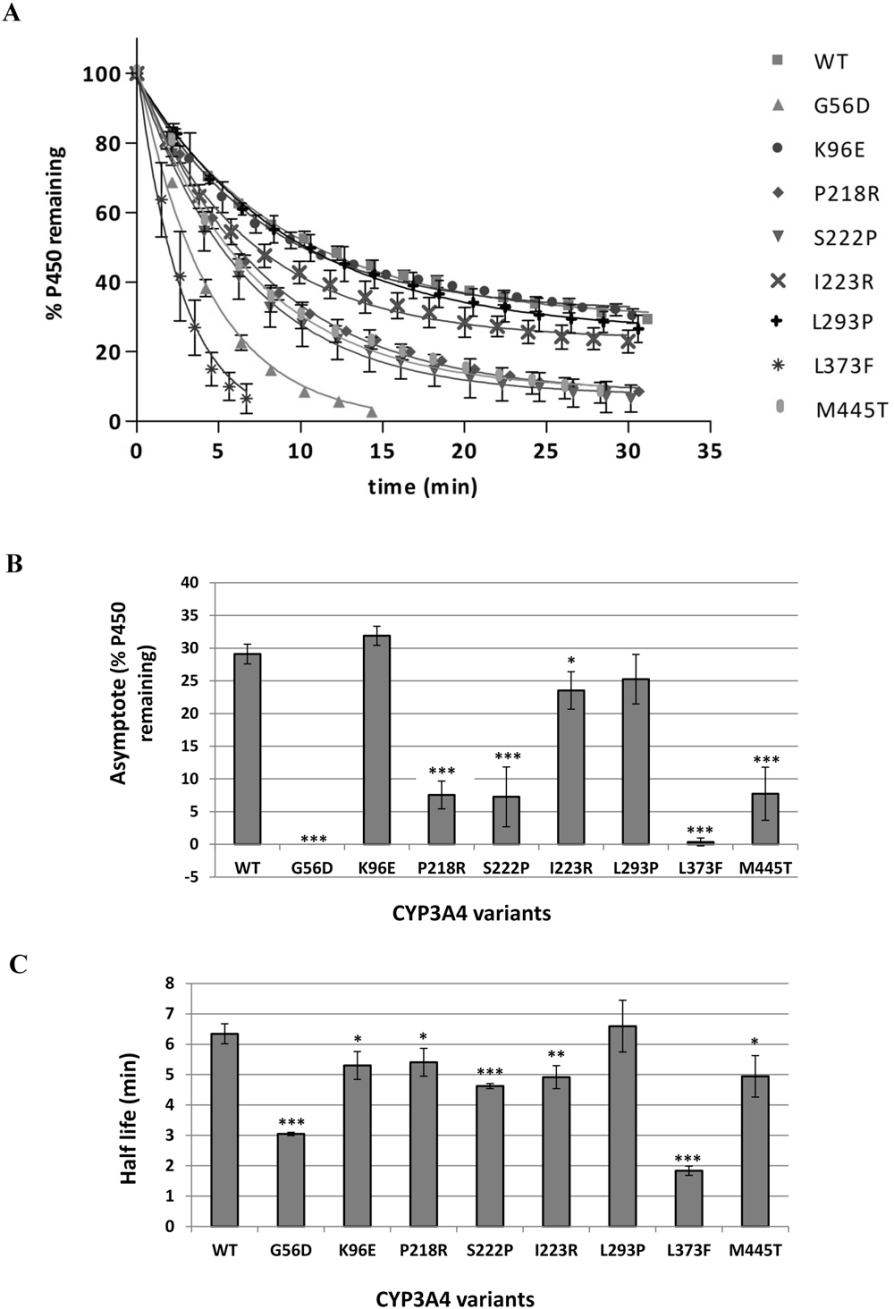
Thermostability of CYP3A4 polymorphic variants at 34 °C. (**A**) Graphs showing the decrease in P450 content at 34 °C over 30 minutes. A one phase exponential decay model was fitted to the data using non-linear regression as described in Methods. (**B**) Comparison of % P450 remaining when the graph reached a low plateau for each variant. (**C**) Comparison of the half-life of each variant at 34 °C. Error bars represent the standard deviation of three replicates. Student t-test was used to determine values significantly different from WT: *P < 0.05, **P < 0.01, ***P < 0.005.

Figure 4 shows the thermostability results for the CYP3A4 WT and variant proteins. Both a one-phase and a two phase-exponential decay model was fitted to the data sets using Graph-Pad Prism software. While the one phase model fitted all the data reasonably well provided the plateau was not constrained to zero, the two-phase model was the preferred fit for WT and variants K96E, S222P, I223R and L293P. The more complex two-phase model gave rate constants with very large confidence intervals that were often inconsistent between replicates. All data sets were therefore analysed using the one phase model, assuming that the contribution of the slower phase to the decrease in P450 content is very small because it has a much longer half-life than the fast phase, resulting in a graph that reaches a low plateau.

To determine the effect of polymorphic variation on CYP3A4 stability, the effect of mutations on the half-life of the unstable sub-population and the effect of mutations on the plateau - the proportion of P450 making up the stable sub-population – were considered. The WT protein had a half-life of 6.3 minutes at 34 °C and reached a plateau with 29% P450 remaining, indicating that approximately one third of the P450 was in the more stable form. The most notable differences in stability compared to WT were observed for variants G56D, L373F, P218R, S222P and M445T (Fig. 4). G56D and L373F plateaued at ~ zero, indicating that these mutations abolish the stable P450 sub-population. They also had the lowest half-lives of all the variants, ~3 and 2 minutes respectively. P218R, S222P and M445T also had a very significant effect on the plateau, with only 7–8% of P450 making up the stable sub-population; however, they only had a moderate effect on the half-life (15–30% decrease) of the unstable sub-population. I223R and K96E mutations had much smaller effects on stability while L293P showed no significant differences compared to WT.

### Comparing the thermostability of CYP2C9 polymorphic variants

**Figure 5:**
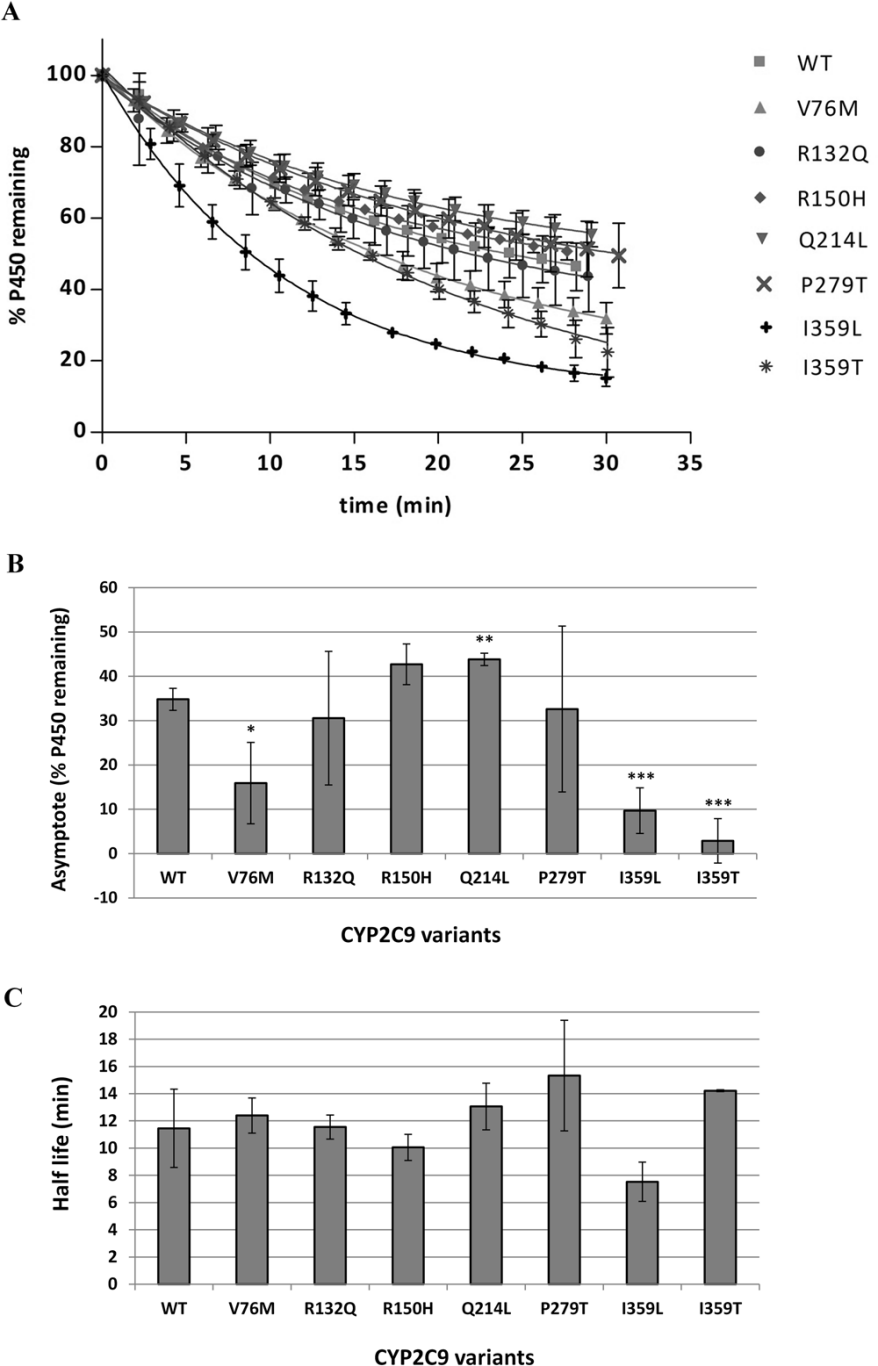
Thermostability of CYP2C9 polymorphic variants at 48 °C. (**A**) Graphs showing the decrease in P450 content at 48 °C over 30 minutes. A one phase exponential decay model was fitted to the data using non-linear regression as described in Methods. (**B**) Comparison of % P450 remaining when the graph reaches a plateau for each variant. (**C**) Comparison of the half-life of each variant at 48 °C. Error bars represent the standard deviation of three replicates. Student t-test was used to determine values significantly different from WT: *P < 0.05, **P < 0.01, ***P < 0.005.

Figure 5 shows the thermostability results for the CYP2C9 WT and variant proteins. The one phase exponential decay model was the preferred model for all the CYP2C9 proteins. The WT protein had a half-life of 11.5 minutes at 48 °C and the graph reached a plateau with 35% P450 remaining. P279T had a half-life of 15.3 minutes (33% higher than WT) and I359L had a half-life of 7.5 minutes (35% lower than WT); however, differences in the half-lives were not significantly different for any of the CYP2C9 variants compared to WT. Q214L, V76M, I359L and I359T did however have a significant effect on the plateau. Q214L had a higher proportion of P450 making up the stable sub-population compared to the WT protein, plateauing with 44% P450 remaining. V76M, I359L and I359T all had adverse effects on the stable P450 populations, with graphs plateauing at 15%, 10% and 3% P450 remaining respectively.

### Size exclusion chromatography to determine quaternary structures of CYP3A4 and CYP2C9 proteins

**Figure 6:**
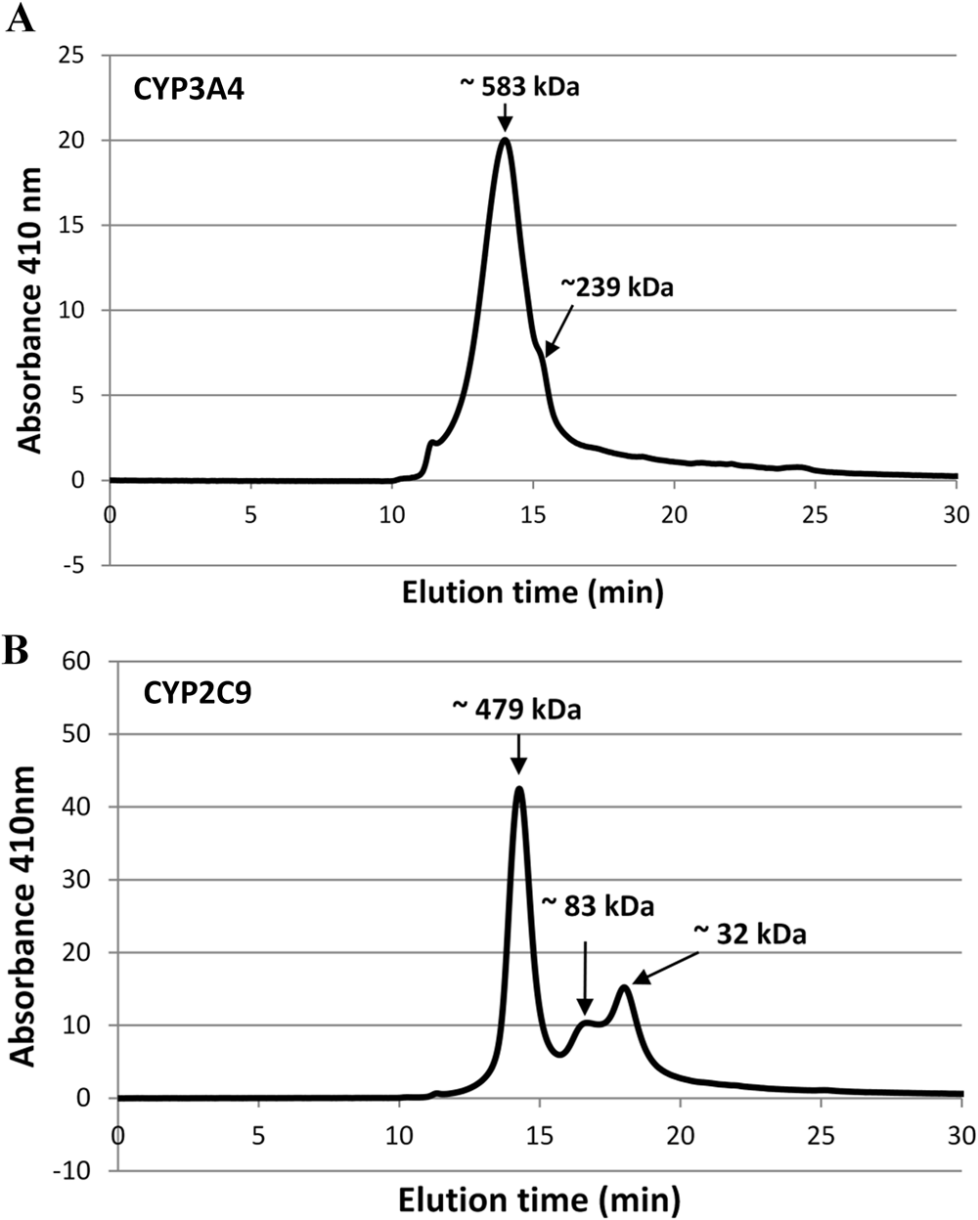
Size exclusion chromatograms for CYP3A4 (**A**) and CYP2C9 (**B**). Elution profiles were measured at 410 nm. The size of the protein corresponding to each peak was estimated using a calibration curve generated from BioRad Standards.

Size exclusion chromatography (SEC) was carried out on CYP3A4 and CYP2C9 WT protein samples to determine whether protein oligomerisation could provide an explanation for the presence of P450 sub-populations with different stabilities in solution. Samples were separated on a BioSep–SEC-S3000 column with exclusion range between 5 kDa and 700 kDa. Figure 6 shows the elution profiles for CYP3A4 and CYP2C9 WT proteins monitored at 410 nm, the maximum absorbance wavelength of the haem-bound protein. The size of the protein corresponding to each peak was estimated using the gel filtration standard calibration curve. The data provides evidence that both isoforms do indeed oligomerise in solution; the major peak for both CYP3A4 (66 kDa in monomeric form) and CYP2C9 (63 kDa in monomeric form) correspond roughly to oligomeric structures made up of 9 and 8 subunits respectively. The major CYP3A4 peak has a shoulder corresponding roughly to a trimeric or tetrameric protein. CYP2C9 shows two secondary peaks (~ 83 and 32 kDa), which do not correspond well to the expected size of a CYP2C9 dimer and monomer, however the columns capability of accurately predicting protein sizes in the lower molecular weight range may be limiting.

## Discussion

In order to test previous *in silico* predictions on the effects of SNPs on CYP450 stability, recombinant soluble CYP2C9 and CYP3A4 WT and variant proteins, lacking their N-terminal membrane binding region, were expressed in *E. coli* and purified from the cell lysate. Holoprotein in the active P450 form was obtained for all proteins. CYP2C9 was much more stable than CYP3A4, to the extent that CYP3A4 had a shorter half-life at 34 °C than CYP2C9 had at 48 °C. The observed differences in stability were consistent with the differences in expression levels and P450:P420 ratios. Notably, the P420 form of the protein was not detected in any of the CYP2C9 samples, while the P420 form was present in all CYP3A4 samples.

Exponential decay curves for both CYP3A4 and CYP2C9 indicated energetically distinct P450 sub-populations that did not interconvert between each other during the course of the assay. In support of this idea, previous published studies examining the binding kinetics of CO to CYP3A4 using flash photolysis have argued that there are two kinetically distinct pools of P450, both in the membrane and in solution^40,41^. Other studies have also observed conformational heterogeneity for CYP2B4^42,43^ and CYP3A4 proteins^44^ using hydrostatic pressure spectroscopy. Elevated pressure typically causes a high-spin to low-spin shift of the haem iron^45–48^, followed by the conversion of P450 to P420; these two pressure-induced processes can occur one after the other^49^ or within overlapping pressure ranges^43,48,50^. Davydov *et al*. observed that only 65 to 70% of CO bound ferrous CYP2B4 in solution was susceptible to a pressure-induced P450 to P420 transition in the absence of detergents, however all P450 could be converted to P420 in the presence of detergent indicating that the observed heterogeneity may be a result of oligomer formation^42^. Similar heterogeneity was also seen for ferric CYP2B4 within proteoliposomal membranes^43^.

In another study it was shown that 70% of CYP3A4 in solution and 50% in microsomes was susceptible to substrate-induced spin shifts and rapid, reversible pressure-induced P450 to P420 conversions^44^. The other fraction of the protein remained predominantly in the low-spin state. In solution, this remaining fraction was subject to slow, irreversible inactivation at high pressures indicating that the pressure induced P450 to P420 conversion of CYP3A4 was biphasic consisting of a fast-reversible phase and a slow-irreversible phase. In microsomes only the fast-reversible phase was observed. A pressure induce high-spin to low-spin shift was also detected in solution phase but not in microsomes, suggesting that the high-spin state is stabilised by interactions with other proteins and lipids within the membrane. Stabilisation of the high-spin state is thought to reflect decreased water accessibility of the haem^51^.

**Figure 7:**
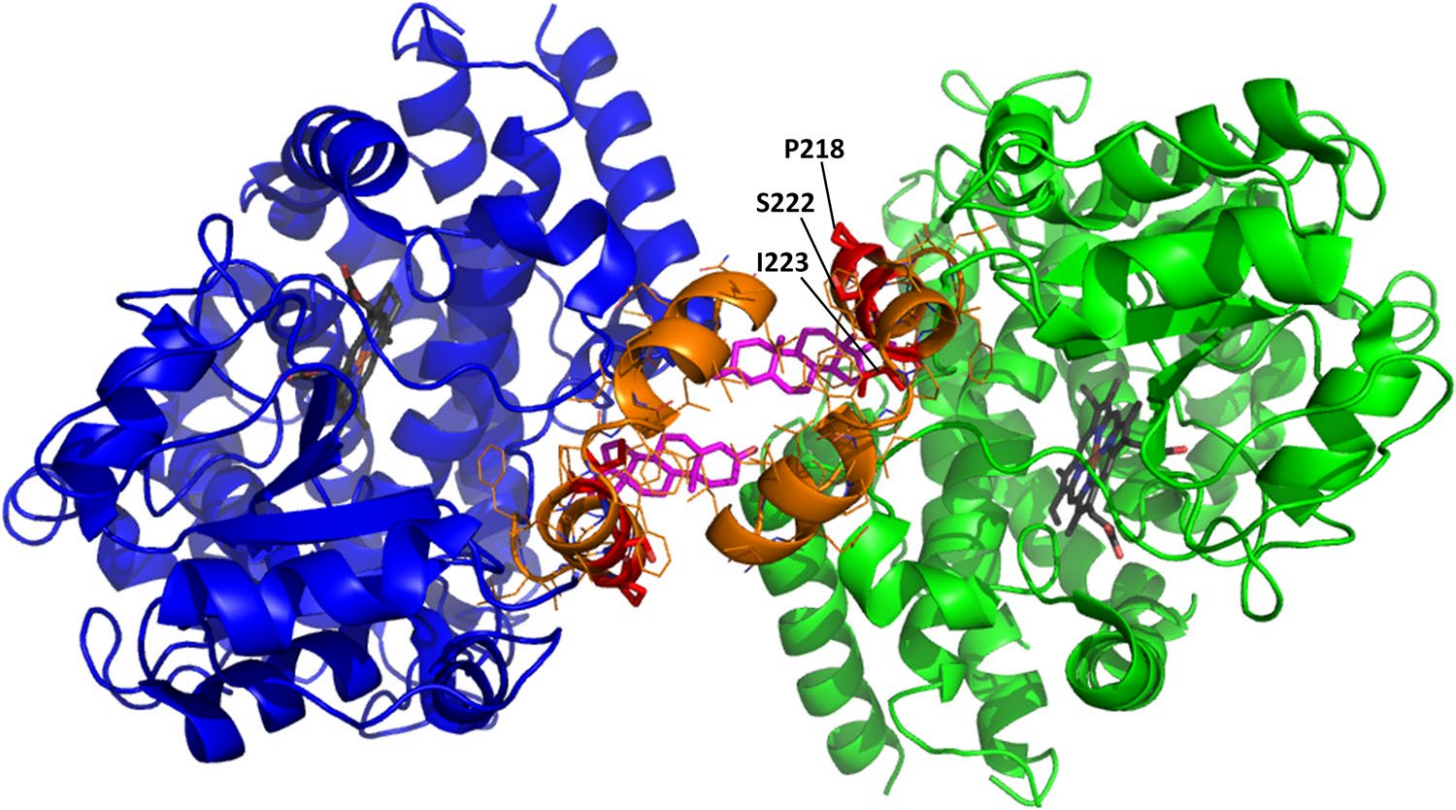
Interaction interface in CYP3A4 dimer. The PDB stucture 1W0F was rendered using PyMOL. The F-G loop is shown in orange and residues Pro 218, Ser 222 and Ile 223 are shown in red. Progesterone molecules bound to the peripheral binding sites at the interface of the molecules are shown in magenta.

Similarly to CYP2B4, the addition of detergents known to break up oligomers has been reported to reduce the heterogeneity of CYP3A4 observed in solution. This led the authors to suggest that the 1:2 conformer distribution in solution is a result of hexameric organisation, where 2 subunits have a different orientation to the other four. Interestingly both CYP2B4 and CYP1A2 have been shown to exist as hexamers in solution^52–55^. In a more recent study, Davydov *et al*. probed the geometry of CYP3A4 oligomers in microsomes using luminescence energy transfer and intermolecular cross-linking experiments^18^. Their results suggest that the most likely size of the CYP3A4 oligomer in solution and in the membrane is a trimer (or multiples of this), involving two different types of interaction interfaces between subunits, one of which is similar to the subunit interface observed in the progesterone-bound CYP3A4 crystal structure (PDB code 1W0F)^56^. An additional progesterone molecule can be observed bound to a peripheral binding situated at the interface of interacting subunits in this structure (Fig. 7). A similar CYP3A4 interface and peripheral binding site is also observed in the crystal structure of CYP3A4 bound to losartan^57^. Further, it has been postulated that CYP3A4 conformational changes induced by allosteric effector molecules (e.g. testosterone) involve ligand binding to this peripheral site and evidence suggests that there is a relationship between oligomerisation and allosteric effectors^58–60^.

In this study we observed similar conformational heterogeneity for both CYP3A4 and CYP2C9 in solution; only 65–70% of the P450 was susceptible to rapid heat-induced P450 to P420 conversion at a constant elevated temperature while the remaining fraction was much more stable, consistent with the literature. Size exclusion chromatography experiments confirmed that both CYP3A4 and CYP2C9 were predominantly in the oligomeric form in solution. The estimated molecular weight of the major CYP3A4 oligomer species were 583 and 239 kDa corresponded most closely to a nonamer and a tetramer, respectively. While nonamers are rarely observed for proteins, a trimer of trimers would agree with Davydov *et al*. work which suggests that CYP3A4 oligomers are made up of trimers and would explain the 1:2 conformer distribution observed for CYP3A4, where 3 of the monomeric subunits are more stable than the other 6. A previous study observed at least three major CYP3A4 oligomers in solution with molecular masses of 215, 290, and 450 kDa using continuous molecular weight distribution analysis^61^.

Our SEC results suggest that CYP2C9 is predominantly in the octomeric form; this does not readily explain the 1:2 conformer distribution observed here. Little is known about the quaternary structure of CYP2C9 in solution or in the membrane. SEC elution times depend both on the size and shape of the protein; the geometry of the oligomer will affect its elution time and hence the predicted molecular weight. Following this, the SEC results here do not conclusively determine the quaternary structure of the protein and further experiments are required to probe the nature of interactions between subunits.

Several of the single amino acid substitutions tested affected the proportion of protein that was sensitive to the rapid heat-induced P450 to P420 conversion and/or affected the half-life of this conversion. Table 1 provides a summary of the results, comparing the previously predicted effects of mutations on the stability of the apo monomeric P450 structure using the *in silico* tool SDM^25^ with the experimentally determined effect of mutations on thermostability.

**Table 1:** Predicted effects of mutations on protein stability versus experimentally determined effects on protein stability.

CYP450 variant	% RSA^Ψ^ of WT residue	Functional region^#^	Predicted effect on stability by SDM^†^	Experimentally determined effect on thermostability
CYP3A4				
G56D (CYP3A4.7)	13.4	SRS1′a	**destabilising**	**decrease**
K96E	60.7	CPR contact	neutral	none*
P218R (CYP3A4.5)	85.8	SRS(2,3)	neutral	decrease
S222P (CYP3A4.2)	52.1	SRS(2,3)	neutral	decrease
I223R	0.0	SRS(2,3)	**destabilising**	**decrease**
L293P (CYP3A4.18)	54.1	start of SRS4	neutral	none
L373F (CYP3A4.12)	13.5	haem contact/SRS5	neutral	decrease
M445T (CYP3A4.3)	39.6	CPR contact	**destabilising**	**Decrease**
CYP2C9				
V76M (CYP2C9.23)	0.1	undefined	neutral	decrease
R132Q (CYP2C9.33)	63.2	CPR contact/SRS2	neutral	none
R150H (CYP2C9.8)	68.3	undefined	neutral	none
Q214L (CYP2C9.28)	6.6	SRS(2,3)	**stabilising**	**increase**
P279T (CYP2C9.29)	90.2	undefined	neutral	none
I359L (CYP2C9.3)	1.7	SRS5	neutral	decrease
I359T (CYP2C9.4)	1.7	SRS5	**destabilising**	**decrease**

^Ψ^RSA refers to the relative solvent accessibility as calculated by (Site Directed Mutator) SDM.

^#^Functional region location based on CYP450 SNP map^25^.

^†^Based on previously reported *in silico* SDM results^25^: destabilising, ∆∆G < −2.0 kcal.mol^−1^; neutral, −2.0 < ∆∆G < 2.0 kcal.mol^−1^; stabilising ∆∆G > 2.0 kcal.mol^−1^.

*K96E showed a small decrease in half-life however this may have been accounted for by the small (although insignificant) increase in the plateau and was therefore not classified as a destabilising mutation.

Mutations in close proximity to the haem-binding region, such as L373F (haem contact) and M445T (predicted to be destabilising by SDM) in CYP3A4, destabilise the haem significantly in both conformers, increasing the proportion of protein sensitive to rapid heat-induced conversion to P420 and decreasing the half-life of this conversion. The most significant effect was observed for L373F, which is in direct contact with the haem: the slow phase was abolished completely and monophasic conversion to P420 occurred at a much more rapid rate. A previous study also reported that L373F showed decreased expression and holoprotein levels compared to WT in an *E. coli* expression system^62^. It was also reported that L373F had a 4-fold higher *K*_m_ for 1′-OH midazolam formation as well as an altered testosterone metabolite profile. This is not surprising since Leu 373, in addition to being a haem contact, falls within substrate recognition site 5.

Met 445 is positioned at the start of the α-helix on the border of the Cys-pocket, two residues away from the haem bound cysteine residue. While this residue does not fall within a substrate recognition site, it is solvent accessible and is predicted to form part of the CYP3A4:CPR interface so any destabilising effects in this region may also alter interactions with CPR. However, studies where the M445T variant was expressed in *E.coli*, quantified using CO spectral binding assays and reconstituted with CPR and lipids, reported no significant difference in turnover numbers for testosterone^62,63^ or chlorpyrifos at the single substrate concentrations tested^63^. The presence of lipids and CPR in their reconstituted reaction mixture may have a stabilising effect on the protein.

CYP3A4 mutation G56D had a similar effect to the L373F mutation; while Gly 56 is not in close proximity to the haem-binding region, it was predicted to be a destabilising mutation by SDM and falls within SRS1′a. A study that expressed the G56D variant in *E.coli* and used the membrane fraction for activity assays found that it had lower *V*_max_ values for midazolam and nifedipine and a 2-fold higher *K*_m_ for testosterone when compared to WT^64^.

For reasons less immediately apparent, P218R and S222P had a significant effect on the thermostability of CYP3A4; P218R and S222P were both predicted to be neutral by SDM and fall within the flexible F-G loop, which is far away from the haem-binding site but forms part of substrate recognition site (2,3) at the proposed interface of the interacting CYP450 subunits, close to the peripheral effector site (Fig. 7). Substrate recognition site (2,3) acts as a “lid” over the active site cavity and is comprised of the F and G helix connected by the F-G loop. The variable F-G loop region is much longer in mammalian CYP450s than in bacterial CYP450s (more than 2 times the length) and is highly flexible, adopting different conformations in different structures of the same isoform. In some CYP450 structures this region is comprised of two short F’ and G’ helixes (e.g. CYP2C9 PDB 1OG2^65^) while in other structures it is a disordered loop region (e.g. CYP2C9 PDB 1R9O^66^). The F-G loop regions forms part of many of the major substrate access channels^36^ and when the enzyme is in a closed conformation comes in close proximity with the active site, thus playing an important role in substrate recognition in mammalian CYP450s^27^. Proline is usually found at the end of an α-helix, acting as a helix breaker; consequently, mutations resulting in the inclusion or exclusion of a proline residue are likely to either stabilise or destabilise the helical conformation of this loop. *In vitro* studies have shown that the S222P mutation causes a 15–70% decrease in *k*_cat_ values for the turnover of testosterone, midazolam and nifedipine^64,67^. The effect of S222P on substrate affinity was reported to be substrate specific and resulted in the following changes to *K*_m_: 3–4 fold higher for nifedipine, 2-fold higher for midazolam and similar for testosterone.

The F-G loop has also been implemented in the dimerization of several mammalian CYP450 isoforms, both in solution and membranes^18,68,69^. In membrane bound CYP450s this loop lies near the membrane or is partially embedded within the membrane. CYP2B4 crystallises as a dimer along a two-fold symmetrical axis, made up of two open structures with the extended F-G loop from one molecule fitting into the open cleft of the second molecule^68^. Close hydrophobic contacts between residues 213–230 and the binding partner were observed, whilst H226 co-ordinated to the haem iron, providing a spectral reporter for dimerization in solution. H226-Fe CYP2B4 dimers were also detected in solution and dimerization could be reversed to form catalytically active monomers.

The dominant effect of P218R and S222P mutations on thermostability is a shift in towards the unstable, heat sensitive conformer, which could be attributed to the disruption of oligomer formation or changes to the oligomer conformation. I223R, another mutation occurring within the F-G loop, causes a significant (P < 0.05) but more moderate shift towards the unstable conformer and a moderate decrease in the half-life of the fast phase. Unlike Pro 218 and Ser 222, which are both solvent exposed residues with side chains orientated towards the bulk solvent, Ile 223 is buried (Table 1, Supplementary Fig. S3) indicating that it is unlikely to interact with other CYP3A4 molecules directly without a substantial change in protein conformation. I223R is predicted to be damaging to protein structure by SDM so the observed effects on thermostability may be caused by destabilisation of the secondary/tertiary structure rather than a direct effect on the quaternary structure.

CYP2C9 variant Q214L, another mutation to a buried residue within the F-G loop and substrate recognition site (2,3), was the only variant predicted to be stabilising by SDM and the only variant that showed a significant increase in thermostability, showing a significant increase in the proportion of stable conformer in solution, but no significant difference in the half-life of the fast phase. *In vitro* studies using mammalian or insect cell expression systems found that Q214L decreases catalytic efficiency by between 45 to 85%^70,71^ confirming the premise that stabilising mutations can also be damaging: increased stability can lead to reduced conformational flexibility that is important for function. A significant decrease in binding affinity was also reported for two of the substrates tested, indicating that this residue plays a more important role in the binding of some substrates than others.

I359L and I359T mutations in CYP2C9 decrease the proportion of protein making up the stable P450 sub-population. Ile 359, the last residue of α-helix K, is buried within the hydrophobic core of the protein and forms part of substrate recognition site 5. SDM correctly predicts that a mutation to threonine at this position will destabilise the protein fold but incorrectly predicts the effect of the conserved mutation to leucine; while isoleucine and leucine are both hydrophobic residues with the same volume and similar properties, the subtle difference between these residues at this position significantly affects the conformation of the CYP2C9 fold. A recently reported crystal structure of the CYP3A4 I359L variant suggests that this mutation has long range effects - transduced along the I-helix - on the orientation of active site residues, altering the binding mode of losartan within the active site compared to the wild-type structure^57^.

I359L and I359T are the non-synonymous amino acid substitutions associated with CYP2C9*3 and CYP2C9*4 alleles, respectively. I359L has long been associated with poor warfarin metabolism and patients with this polymorphism require lower warfarin doses as they are at higher risk of bleeding complications when using this anticoagulant^72^. Several *in vitro* studies, most of which are reports on I359L, show that mutations at this position lead to a large decrease in catalytic activity and an increase in *K*_m_ for some but not all substrates tested^72–79^.

CYP2C9 variant V76M also showed significantly less P450 in the stable sub-population than observed for the WT protein but was not predicted to be a destabilising mutation by SDM. V76 is a buried residue within strand 2 of β-sheet 1 and does not fall within any know functional region of the protein. While Val 76 is position roughly 13 Å from the bound haem, the model of the CYP2C9 V76M variant used to generate the SDM data suggests that the substitution of valine for the bulkier methionine residue at this position has a subtle long-range effect on the side chain position of His 368, which interacts with the haem propionate via a salt bridge. While this mutation is not predicted to affect the stability of the apoprotein, it is conceivable that these changes disrupt the correct positioning of the haem ion in the holoprotein, hence explaining the apparent discrepancy between the *in silico* analysis and the experimental results.

While CYP2C9 R132Q and R150H showed no significant effect on the CYP450 stability in agreement with the *in silico* predictions, these SNPs have both been reported to affect catalytic activity. CYP2C9 R132Q, despite showing similar holo P450 levels in a baculovirus-insect cell system, showed ~90% reduction in catalytic activity towards diclofenac, losartan and glimepiride^71,80^. Arg 132 is situated in the loop region between helix C and D and is highly conserved in the CYP2C family. It is predicted to form part of the charge cluster responsible for electrostatic interactions with CPR^34^ and consequently the reduction in catalytic activity has largely been attributed to altered binding affinity for its redox partner. CYP2C9 R150H does not fall within any of the known functional regions. Despite this, it has been reported to affect CYP2C9 function *in vivo* and *in vitro*. The CYP2C9*8 R150H allele commonly occurs in African Americans and has been associated with decreased warfarin and phenytoin metabolism^81,82^. In addition, an *in vitro* study found that this variant had improved catalytic activity and a 2-fold lower *K*_m_ for tolbumide, resulting in a 2-fold increase in catalytic efficiency^83^, suggesting that this mutation might have substrate specific effects on metabolism. This mutation is situated on the surface of the protein, outside the cluster of charged residues responsible for CPR binding, but it has been proposed that it could affect interactions with CPR^83^.

In conclusion, 10 out of the 15 variants proteins tested had significantly altered thermostability compared to the corresponding WT protein. Buried SNPs in close proximity to the haem (e.g. CYP3A4 L373F) as well as SNPs further away from the haem-binding site (e.g. CYP3A4 G56D) - including residues in solvent accessible, variable flexible regions predicted to be neutral (e.g. CYP3A4, S222P and P218R) - had significant effects on thermostability of the P450 form. The results suggest that the observed effects on protein thermostability in solution may result from changes in the haem-binding environment, alterations in oligomer formation or destabilisation of the protein fold. Based on these *in vitro* results, *in silico* predictions by SDM had a specificity of 100% (5/5) and a sensitivity of 50% (5/10). SDM’s low sensitivity is expected since effects on haem binding and quaternary structure are not accounted for by SDM: of the 5 false negatives, as predicted by SDM, one (L373F) is a haem contact and two may alter oligomer formation (P218R, S222P); the other two variants were either incorrectly predicted by SDM or have long range effects on oligomer formation, CYP450 conformation or haem binding, without significantly disrupting the stability of the protein fold.

This work provides new insight into structure-function relationships in CYP3A4 and CYP2C9 and demonstrates the utility of *in silico* approaches such as SDM combined with the CYP450 functional map^25^ for predicting the effects of mutations on CYP450 stability and prioritising SNPs for experimental testing. There is a growing body of evidence indicating that homo- and hetero-oligomer formation plays a key role in CYP450 regulation in membranes^18,19,84^. The study shows that the effect of mutations on P450:P450 interactions may be an important consideration when predicting the effect of polymorphic variation on drug metabolism.

## Methods

### Expression Plasmids: CYP3A4 and CYP2C9

pBJW102.2 vectors containing the N-terminal truncated CYP3A4 and CYP2C9 cDNA sequences were a gift from Procognia Ltd, Maidenhead, UK. The pBJW102.2 parent vector was originally created by cloning the BCCP domain of the *E.coli* AccB enzyme (amino acids 74–156) and a glycine-serine linker sequence in frame, 3′ to the His-6 tag of the commercially available pQE-80L *E.coli* expression vector (Qiagen, USA). The translated CYP3A4 and CYP2C9 proteins had a 24 amino acid and 28 amino acid deletion at the N-terminus respectively.

### Construction of polymorphic mutations in CYP3A4 and CYP2C9

Expression plasmids for 8 CYP3A4 and 7 CYP2C9 polymorphic variants were generated from the pBJW102.2 + CYP3A4 and the pBJW102.2 + CYP2C9 wild-type expression plasmids by inverse PCR using the 5′phosphorylated primers listed in Supplementary Table S1. The mutated plasmids were sequenced by Inqaba Biotechnical Industries.

### Recombinant protein expression and purification

#### Expression

Terrific Broth medium supplemented with trace elements (250 µM FeCl_3_, 24 µM ZnCl_3_, 21 µM CoCl_2_, 21 µM Na_2_MoO_4_, 0.15 µM CaCl_2_, 20 µM H_3_BO_3_, 10 µM CuCl_2_, 0.03% HCl), 1 mM thiamine and 100 µg/ml ampicillin was inoculated with an overnight starter culture of *E.coli DH5α* containing the expression plasmid. Cultures were incubated at 37 °C with shaking at 160 rpm until the cell density reached OD_600_ 0.4–0.6. δ-aminolevulinic to a final concentration of 0.5 mM was added to facilitate haem synthesis and cultures were incubated at 30 °C with shaking at 160 rpm for a further 30 minutes. 1 mM IPTG was added to induce protein expression and 50 µM biotin was added to facilitate biotinylation of the BCCP-tag. Expression was carried out at 30 °C with shaking at 120 rpm for 18 h. Cells were harvested by centrifugation at 4 °C, washed in 1 × phosphate-buffer saline and stored at −20 °C.

#### Protein extraction and purification

Cell pellets were resuspended in 20 mM phosphate buffer, pH 7.4 containing 20% glycerol (v/v) and 10 mM β-mercaptoethanol. 1.5 mg/ml lysozyme, 80 U/ml DNase I, 0.5 mM CaCl_2_ and 2.5 mM MgCl_2_ was added and the cell suspensions were left on ice with gentle shaking for 30 minutes. 0.15% IgePal CA-630, 0.5% CHAPS and 0.1% Triton X-100 was added and cell suspensions were left on ice with gentle shaking for a further 30 minutes. The crude soluble lysates were then separated from the cell debris by centrifugation at 10 000 × g at 4 °C for 30 minutes and expression was confirmed by western blot analysis using streptavidin-horseradish peroxidase (strep-HRP). His-tag purification was carried out using Protino Ni-TED packet columns (Macherey-Nagel, Germany), according to manufacturer’s instructions. The eluted samples were concentrated to <700 µl using Amicon Ultra 15 ml centrifugal filters (10 K MWCO; Merk Millipore Ltd, Ireland) by centrifugation at 4000 × g at 4 °C for ~45 minutes. Protein samples were buffer exchanged from the Ni-TED elution buffer into storage buffer (20 mM potassium phosphate, pH 7.4; 20% glycerol (v/v); 0.2 mM EDTA; 1 mM DTT) using Zeba desalting columns (7 K MWCO; Pierce, USA). Total protein concentration was determined using the Bradford assay and protein was visualised on Coomassie-Blue stained SDS-PAGE gels.

### Specific protein quantification: Carbon monoxide P450 spectral Assays

CYP3A4 and CYP2C9 holo P450 protein content in the enriched protein samples was determined using carbon monoxide spectral assays^39^. CYP450 protein samples were diluted in storage buffer to a concentration of 1 mg/ml and divided into two cuvettes (one reference and one sample cuvette). The cuvettes were placed into a Varian Cary 50 conc UV Visible spectrophotometer (Varian, Australia) and a baseline reading between 400 and 500 nm was recorded. Carbon monoxide (Speciality Gasses, SA) was bubble into the bottom of the sample cuvette (~60 bubbles at a rate of 1 bubble per second). A small spatula tip (~1 mg) of sodium dithionite (Na_2_S_2_O_4_, Sigma-Aldrich, Germany) was added to each cuvette. Cuvettes were covered with parafilm and inverted several times to dissolve the sodium dithionite. The cuvettes were then placed back inside the spectrophotometer and spectra between 400 and 500 nm were measured several times until the peak at 450 nm stopped increasing.

Readings from the final spectra were used to calculate the amount of P450 and P420 protein present in the sample using the following equations:

11 $$[{({{\rm{\Delta }}{\rm{A}}}_{450}-{{\rm{\Delta }}{\rm{A}}}_{420})}_{{\rm{observed}}}-{({{\rm{\Delta }}{\rm{A}}}_{450}-{{\rm{\Delta }}{\rm{A}}}_{420})}_{{\rm{baseline}}}]/0.091={\rm{nmol}}\,{\rm{P}}450\,{\rm{per}}\,{\rm{ml}}$$

12 $$\begin{array}{c}[{({{\rm{\Delta }}{\rm{A}}}_{420}-{{\rm{\Delta }}{\rm{A}}}_{490})}_{{\rm{observed}}}-{({{\rm{\Delta }}{\rm{A}}}_{420}-{{\rm{\Delta }}{\rm{A}}}_{490})}_{{\rm{theoretical}}}\\ \,\mbox{--}[{({{\rm{\Delta }}{\rm{A}}}_{420}-{{\rm{\Delta }}{\rm{A}}}_{490})}_{{\rm{baseline}}}]/0.110={\rm{nmol}}\,{\rm{of}}\,{\rm{P}}420\,{\rm{per}}\,{\rm{ml}}\end{array}$$

where

13 $${({{\rm{\Delta }}{\rm{A}}}_{420}-{{\rm{\Delta }}{\rm{A}}}_{490})}_{{\rm{t}}{\rm{h}}{\rm{e}}{\rm{o}}{\rm{r}}{\rm{e}}{\rm{t}}{\rm{i}}{\rm{c}}{\rm{a}}{\rm{l}}}=({\rm{n}}{\rm{m}}{\rm{o}}{\rm{l}}\,{\rm{P}}450\,{\rm{p}}{\rm{e}}{\rm{r}}\,{\rm{m}}{\rm{l}}\,{\rm{f}}{\rm{r}}{\rm{o}}{\rm{m}}\,[1.1])\times (-0.041)$$

### Thermostability assays

CYP450 protein samples were diluted in storage buffer to a concentration of ~1 µM P450. The CO P450 spectral assay was carried out as described above. After the addition of sodium dithionite, the cuvettes were incubated at room temperature for 5 minutes to allow time for the P450 peak to reach a maximum prior to starting the thermostability assay. The cuvettes were then placed back into the spectrophotometer maintained at a constant temperature of 34 °C (for CYP3A4 samples) or 48 °C (for CYP2C9 samples) by circulating water from a heated water bath through the cell holder. Several readings were taken over the first 30 seconds to ensure the P450 peak had stopped increasing and to allow time for the cuvette to equilibrate to the new temperature. The reading with the highest P450 peak was taken as time zero and following this, readings were taken at 2 minute intervals for 30 minutes. Assays were carried out in triplicate. The amount of P450 in each sample at each time point was calculated using the equations above.

Time vs. % P450 remaining was plotted for each replicate. Graph-Pad Prism software (San Diego, USA) was used to fit both one phase (Equation 2) and two phase (Equation 3) exponential decay models to the data sets using non-linear regression. The plateau was constrained to values greater than or equal to zero. A comparison of the two models was performed in Prism using the F-test. The model that fitted better to 2 or more of the 3 replicate data sets was considered the preferred model for the given variant protein. Reported parameters are however all based on the one phase model.

2 $$Y=({Y}_{0}-Plateau){e}^{-Kx}+Plateau$$

Where Y_0_ is the Y value when X (time) is zero (*i.e*. 100% P450), Plateau is the Y value at infinite X (time), K is the rate constant (min^−1^) and half-life = ln(2)/K.

3 $$Y=Plateau+SpanFas{t}^{\ast }{e}^{-Kfas{t}^{\ast }X}+SpanSlo{w}^{\ast }{e}^{-Kslo{w}^{\ast }X}\,$$

Where SpanFast + SpanSlow + Plateau = Y_0_, Kfast and Kslow are the two rate constants (min^−1^), half-life(fast) = ln(2)/Kfast and half-life(slow) = ln(2)/Kslow.

### Size exclusion chromatography

CYP3A4 and CYP2C9 WT protein samples were separated using the BioSep-SEC-S3000 column with exclusion range of 5 kDa to 700 kDa. Chromatography was carried out at room temperature at a flow rate 0.5 ml/min and pressure ~28 bars. The column was washed with distilled water and equilibrated with 100 mM potassium phosphate buffer, pH 7.4 until a steady baseline reading was obtained. The column was calibrated using a 10 µl injection of Bio-rad gel filtration standard (Catalog#151–1901). 20 µl of CYP450 protein sample in storage buffer (~2 mg/ml total protein concentration) was injected onto the column. The UV absorbance was monitored at 410 nm, the maximum absorbance of the haem bound protein, using the Agilent 1260 infinity HPLC system and the molecular weight of the protein corresponding to each peak in the chromatogram was estimated using the Bio-rad gel filtration standard calibration curve.

### Data availability statement

All data generated or analysed during this study are included in this published article (and its Supplementary Information files) or are available from the corresponding author on reasonable request.

## Electronic supplementary material

Supplementary Information
