# Supplementary material for: Effects of polymorphic variation on the thermostability of heterogenous populations of CYP3A4 and CYP2C9 enzymes in solution
Source: Sci Rep. 2018 Aug 8;8:11876. doi: 10.1038/s41598-018-30195-1 (PMC6082832; doi:10.1038/s41598-018-30195-1)
Supplement: Supplementary file 1 — Supplementary Information [file 41598_2018_30195_MOESM1_ESM.pdf]

Effects of polymorphic variation on the thermostability of heterogenous populations of CYP3A4 and CYP2C9 enzymes in solution

Lauren B Arendse[1] and Jonathan M Blackburn[1]*

From the [1]Institute for Infectious Disease & Molecular Medicine, Department of Integrative Biomedical Sciences, Faculty of Health Sciences, University of Cape Town, Observatory 7925, South Africa

***To whom correspondence should be addressed: Jonathan M Blackburn, Institute for Infectious Disease & Molecular Medicine, Department of Integrative Biomedical Sciences, Faculty of Health Sciences, University of Cape Town, Observatory 7925, South Africa; jonathan.blackburn@uct.ac.za; Tel: +27 21 406 6071

SUPLEMENTARY INFORMATION

Table S1 Primers used to introduce single point mutations into CYP3A4 and CYP2C9 genes within the pBJW102.2 vector by inverse PCR

Mutation	Change	Forward Primer (5'-3')	Reverse Primer (5'-3')	T_A (°C)
**3A4**				
**G56D**	224 G>A	ACTTTTGTATGTTTGACATGGAATG	CCTTATGGTAGGACAAAATATTTCC	51
**K96E**	343 A>G	GAAGAATGTTATTCTGTCTTCAC	CACTAGCACTGTTTTGATCATGTC	54
**P218R**	710 C >G	GATTCTTTCTCTCAATAACAGTCT	GATCCAAAAAATCAAATCTTAAAAG	47
**S222P**	721 T>C	CCAATAACAGTCTTTCCATTCCTC	GAGAAAGAATGGATCCAAAAAATC	51
**I223R**	725 T>G	GAACAGTCTTTCCATTCCTCATC	TTGAGAGAAAGAATGGATCCAAA	54
**L293P**	935 T>C	CGGAGCTCGTGGCCCAATCAATTA	GATCGGACAGAGCTTTGTGGGACT	62
**L373F**	1174 C>T	TTTGAGAGGGTCTGCAAAAAAGATG	TCTCATAGCAATTGGGAATAATCTG	55
**M445T**	1391 T>C	CGAGGTTTGCTCTCATGAACATG	TGCCAATGCAGTTTCTGGGTCCAC	70
**2C9**				
**V76M**	271 G>A	ATGCTGCATGGATATGAAGCAGTG	CACTATGGGTTTCAGGCCAAAATA	57
**R132Q**	440 G>A	AGAATTTTGGGATGGGGAAGAGG	GCAGCGTCATGAGGGAGAAACG	62
**R150H**	494 G>A	ACTGCCTTGTGGAGGAGTTGAGA	GGGCTTCCTCTTGAACACGGTC	62
**Q214L**	686 A>T	TGATCTGCAATAATTTTTCTCCTA	GGATCCAGGGGCTGCTCAAAAT	57
**P279T**	880 C>A	ACATCTGAATTTACTATTGAAAGC	TTGGTTGTGCTTTTCCTTCTCCA	57
**I359L**	1120 A>C	CTTGACCTTCTCCCCACCAGCCT	GTATCTCTGGACCTCGTGCACCA	64
**I359T**	1121 T>C	CTGACCTTCTCCCCACCAGCCT	TGTATCTCTGGACCTCGTGCACC	64

All primers were 5'phosphorylated. Annealing temperatures (T_A) used for each set of primers are shown in the last column.

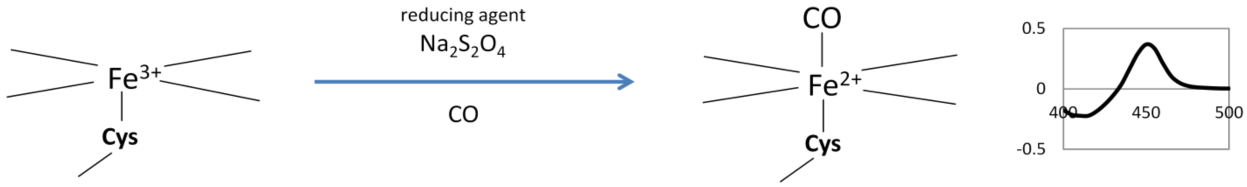

Figure S1. Schematic of CO P450 spectral assay. The ferric haem ion is converted to a ferrous ion in the presence of a reducing agent, followed by the co-ordination of CO at the axial position leading to an absorption peak at 450 nm.

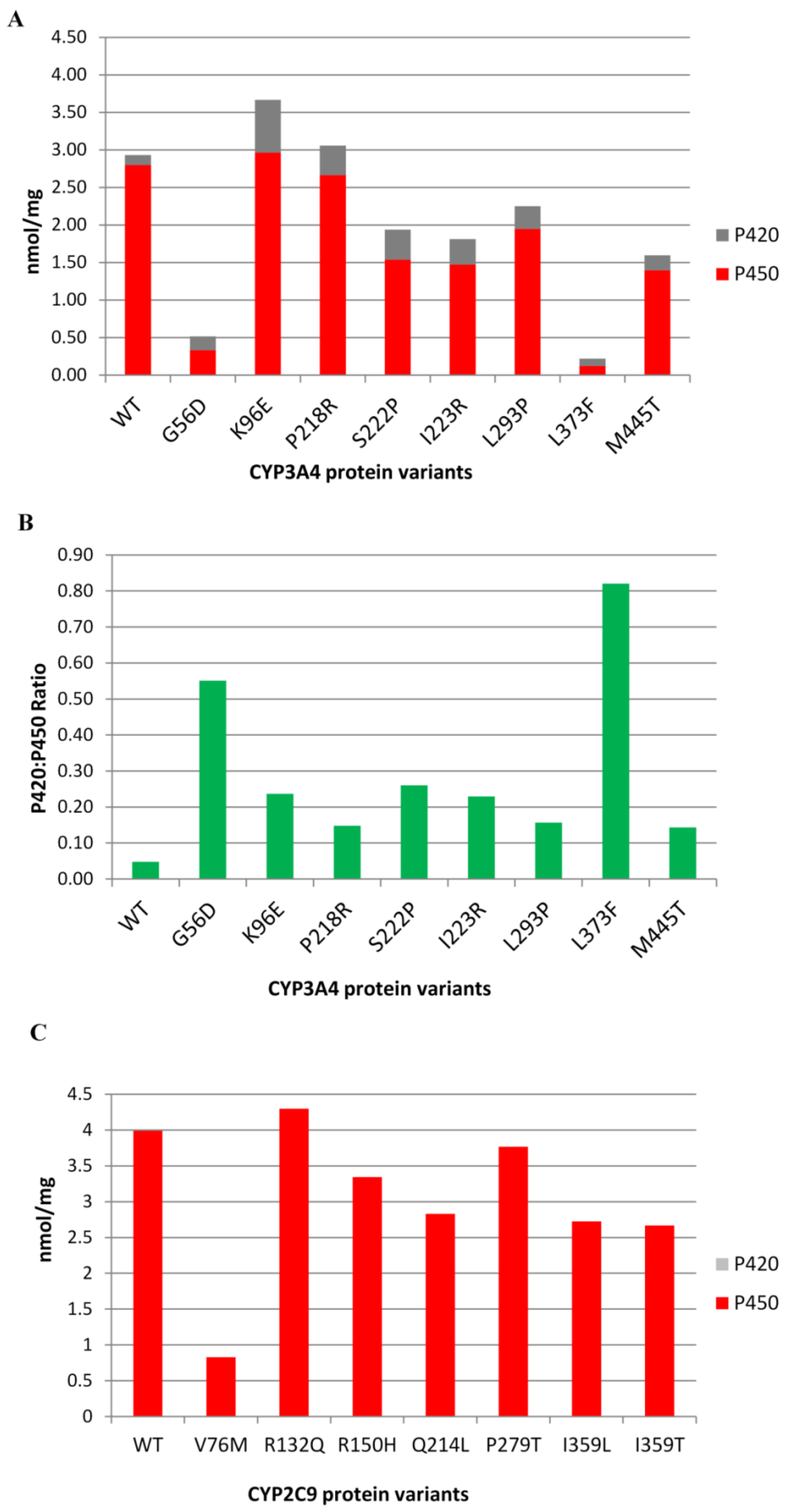

Figure S2. P450 and P420 protein content in CYP3A4 and CYP2C9 protein samples measured by CO P450 spectral assays. (A) nmols P450 and P420 per mg of total protein for CYP3A4 wild-type and variant samples. (B) P420:P450 ratios for CYP3A4 wild-type and variant samples. (C) nmols P450 per mg of total protein for CYP2C9 wild-type and variant samples. Note: P420 protein was not detected in any of the CYP2C9 protein preparations.

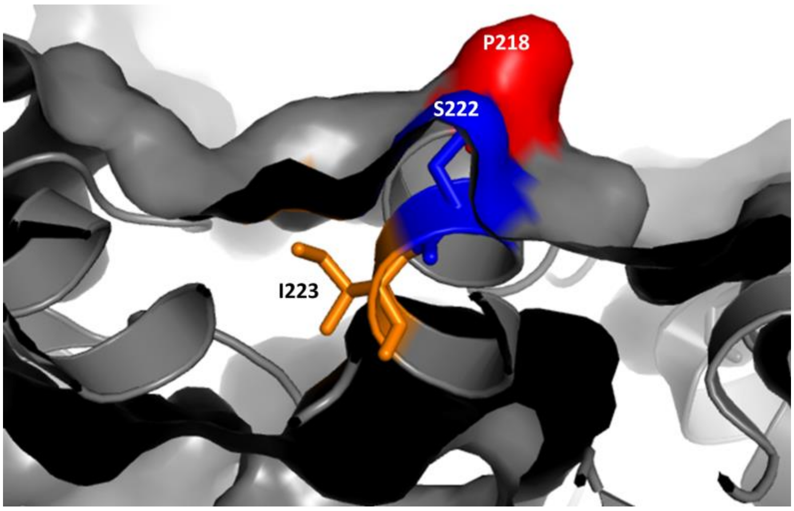

Figure S3. Surface accessibility of CYP3A4 residues P218, S222 and I223. PDB structure 1TQN was rendered using PyMOL, illustrating the surface accessibility of residues P218 (red), S222 (blue) and I223 (yellow) within the F' helix. P218 and S222 side chains form part of the surface of the molecule whereas I223 is buried beneath the surface.
